# Supplementary material for: Evolutionary and Structural Analysis of PP16 in Viridiplantae
Source: Int J Mol Sci. 2024 Feb 29;25(5):2839. doi: 10.3390/ijms25052839 (PMC10931576; doi:10.3390/ijms25052839)
Supplement: Supplementary file 1 [file ijms-25-02839-s001.zip › ijms-2865055-supplementary.pdf]

International Journal of
Molecular Sciences

Evolutionary and structural analysis of PP16 in Viridiplantae

Domingo Jiménez-López[1], Beatriz Xoconostle-Cázares[1], Berenice Calderón-Pérez[1], Brenda Yazmín Vargas-Hernández[1], Leandro Alberto Núñez-Muñoz[1], José Abraham Ramírez-Pool[1] and Roberto Ruiz-Medrano[1]*

* Correspondence: rmedrano@cinvestav.mx

Supplementary Materials

Table S1. Selected plant species and their PP16 homologs retrieved from the Phytozome database. Chlorophytes (green algae) PP16-related proteins are also listed.

No.	Category	Abbreviation|Species	PP16 homologs
1	Basal embryophytes	ppp|*Physcomitrella patens*	112279523_ERG1-like<br>112289355_ERG3-like
2		smo|*Selaginella moellendorffii*	SELMODRAFT_148785<br>SELMODRAFT_229026
3	Basal angiosperm	atr|*Amborella trichopoda* (v1.0)	scaffold00009.330<br>scaffold00009.331
4	Monocots	spo|*Spirodela polyrhiza* (v2.0)	Spipo23G0016400<br>Spipo23G0016300<br>Spipo16G0035500
5		osa|*Oryza sativa* (v7.0)	LOC_Os01g62430.3_OsERG1_RPP17<br>LOC_Os04g44870.1_OsERG3_RPP16<br>LOC_Os04g58570.1<br>LOC_Os02g42710.1
6		pvi|*Panicum virgatum* (v5.1)	Pavir.5KG621600.2.p<br>Pavir.5NG582800.1.p<br>Pavir.1NG368400.1.p<br>Pavir.1KG408900.1.p<br>Pavir.7NG338700.1.p<br>Pavir.7NG444500.3.p<br>Pavir.7KG372100.3.p

7		sbi|*Sorghum bicolor* (v3.1.1)	Sobic.003G352400.1<br>Sobic.004G228300.1<br>Sobic.006G157900.1<br>Sobic.006G271300.1
8		zma|*Zea mays* (v4.0)	Zm00001d042909_P001<br>Zm00001d051249_P003<br>Zm00001d002850_P001<br>Zm00001d026642_P001
9	Eudicots	sly|*Solanum lycopersicum* (ITAG3.2)	Solyc04g011540.3.1<br>Solyc10g018060.2.1<br>Solyc08g080680.3.1
10		stu|*Solanum tuberosum* (v6.1)	Soltu.DM.04G007530.1<br>Soltu.DM.10G007190.1<br>Soltu.DM.08G027310.1
11		egr|*Eucalyptus grandis* (v2.0)	Eucgr.J00003.1<br>Eucgr.E00562.1
12		vvi|*Vitis vinifera* (v2.1)	VIT_213s0019g01490.1<br>VIT_208s0007g00430.1<br>VIT_202s0025g04350.1
13		pop|*Populus trichocarpa* (v4.1)	Potri.010G203000.2.p<br>Potri.010G203100.1.p<br>Potri.002G155600.1.p
14		rco|*Ricinus communis* (v0.1)	27383.m000160<br>30190.m011166<br>30174.m008982
15		csi|*Citrus sinensis* (v1.1)	g035563m_ERG1_CsPP16-1_XP_006486477.1<br>g046962m_At1g63220-like_CsPP16-2_XP_006477594.2<br>g037768m_ERG3-like_isoform-1_CsPP16-3_XP_006491029.1
16		ccl|*Citrus clementina* (v1.0)	Ciclev10032979m<br>Ciclev10024188m<br>Ciclev10022928m
17		cpa|*Carica papaya* (ASGPB v0.4)	1.414<br>1.415<br>18.207
18		tca|*Theobroma cacao* (v2.1)	Thecc.10G040600.1.p<br>Thecc.03G274700.1.p<br>Thecc.01G359300.1.p
19		aly|*Arabidopsis lyrata* (v2.1)	AL5G36620.t1<br>AL2G12160.t1
20		ath|*Arabidopsis thaliana* (TAIR10)	AT3G55470.1_AtPP16-1<br>AT1G63220.1_AtPP16-2

21		bra|*Brassica rapa* (FPsc v1.3)	Brara.D00394.1<br>Brara.I03796.1<br>Brara.I01220.1
22		esa|*Eutrema salsugineum* (v1.0)	Thhalv10010786m<br>Thhalv10023745m
23		csa|*Cucumis sativus* (v1.0)	Cucsa.010870.1<br>Cucsa.010880.1<br>Cucsa.302880.1
24		cmax|*Cucurbita maxima*	Q9ZT47.3_PP16-1*<br>AAY96411.1_PP16-2*<br>XP_022991040.1_PP16-1-like_isoform-X1*<br>XP_022992698.1_ERG1-like<br>XP_022996373.1_ERG3-like
25		cmos|*Cucurbita moschata*	ABK41006.1_PP16-1<br>ABK41007.1_PP16-2<br>XP_022921678.1_PP16-2-like_isoform-X1<br>XP_022953694.1_PP16-1-like_isoform-X1<br>XP_022938500.1_ERG1-like<br>XP_022957957.1_ERG3-like
26		gmx|*Glycine max* (Wm82.a4.v1)	Glyma.02G247900.3.p<br>Glyma.11G219000.1.p<br>Glyma.18G038400.1.p<br>Glyma.14G068400.1.p<br>Glyma.02G248000.1.p<br>Glyma.02G248100.1.p<br>Glyma.03G015400.1.p<br>Glyma.07G075300.2.p<br>Glyma.18G224800.1.p<br>Glyma.08G011900.1.p<br>Glyma.05G204900.1.p
27		pvu|*Phaseolus vulgaris* (v2.1)	Phvul.008G232800.1<br>Phvul.008G232700.1<br>Phvul.008G232600.1<br>Phvul.002G286900.1<br>Phvul.010G093800.1<br>Phvul.008G073400.1
28		pper|*Prunus persica* (v2.1)	Prupe.2G236500.1<br>Prupe.6G256400.1<br>Prupe.2G158500.1<br>Prupe.5G094600.1<br>Prupe.2G158700.1<br>Prupe.2G158800.1<br>Prupe.5G094700.1

29	Green algae	cre|*Chlamydomonas reinhardtii* (v5.5)	Cre01.g015500.t1.2<br>Cre02.g110350.t1.2<br>Cre17.g723700.t1.1<br>Cre12.g485200.t1.1<br>Cre11.g467599.t1.1<br>Cre11.g467594.t1.1<br>Cre11.g467593.t1.1<br>Cre11.g467601.t1.1<br>Cre11.g467595.t1.1<br>Cre06.g305750.t1.2<br>Cre02.g074900.t1.1
30		vca|*Volvox carteri* (v2.1)	Vocar.0007s0408.1.p<br>Vocar.0013s0032.1.p<br>Vocar.0009s0235.1.p<br>Vocar.0001s1625.1.p<br>Vocar.0015s0039.1.p<br>Vocar.0009s0192.1.p<br>Vocar.0004s0220.1.p
31		csl|*Coccomyxa subellipsoidea* (C-169 v2.0)	63427<br>56339
32		mpp|*Micromonas pusilla* (CCMP1545 v3.0)	36336<br>57933<br>63907<br>58763
33		mis|*Micromonas* sp. (RCC299 v3.0)	59753<br>61618<br>59622
34		olu|*Ostreococcus lucimarinus* (v2.0)	8959<br>9049

* These sequences were used as query for identification and retrieval of PP16 homologs or related proteins.

Table S2. C2 domain of the PP16 proteins in Viridiplantae species. The C2 domains of PP16 related proteins in green algae species are included. The data were obtained from the Kyoto Encyclopedia of Genes and Genomes database (https://www.genome.jp/tools/motif/).

Query	Pfam	Position (E-value)	Description
cmax|Cucurbita_maxima_Q9ZT47.3_PP16-1	C2	5..104(1.4e-15)	PF00168, C2 domain
cmax|Cucurbita_maxima_AAY96411.1_PP16-2	C2	5..110(6.3e-18)	PF00168, C2 domain
cmax|Cucurbita_maxima_XP_022991040.1_PP16-1-like_isoform-X1	C2	4..98(5e-19)	PF00168, C2 domain
cmax|Cucurbita_maxima_XP_022991040.1_PP16-1-like_isoform-X1	B9-C2	34..95(0.096)	PF07162, Ciliary basal body-associated, B9 protein
cmax|Cucurbita_maxima_XP_022992698.1_ERG1-like	C2	4..100(7e-20)	PF00168, C2 domain
cmax|Cucurbita_maxima_XP_022996373.1_ERG3-like	C2	4..94(3.4e-20)	PF00168, C2 domain
cmos|Cucurbita_moschata_ABK41006.1_PP16-1	C2	4..109(2e-17)	PF00168, C2 domain
cmos|Cucurbita_moschata_ABK41007.1_PP16-2	C2	4..110(2.4e-18)	PF00168, C2 domain
cmos|Cucurbita_moschata_ABK41007.1_PP16-2	DUF6402	37..75(0.076)	PF19940, Family of unknown function (DUF6402)
cmos|Cucurbita_moschata_XP_022921678.1_PP16-2-like_isoform-X1	C2	4..109(1e-16)	PF00168, C2 domain
cmos|Cucurbita_moschata_XP_022953694.1_PP16-1-like_isoform-X1	C2	4..98(2.4e-19)	PF00168, C2 domain
cmos|Cucurbita_moschata_XP_022953694.1_PP16-1-like_isoform-X1	B9-C2	33..94(0.046)	PF07162, Ciliary basal body-associated, B9 protein
cmos|Cucurbita_moschata_XP_022938500.1_ERG1-like	C2	4..100(3.9e-19)	PF00168, C2 domain
cmos|Cucurbita_moschata_XP_022938500.1_ERG1-like	NT-C2	37..127(0.11)	PF10358, N-terminal C2 in EEIG1 and EHBP1 proteins
cmos|Cucurbita_moschata_XP_022957957.1_ERG3-like	C2	4..94(3.4e-20)	PF00168, C2 domain
ppp|Physcomitrium_patens_112279523_ERG1-like	C2	4..94(4.4e-17)	PF00168, C2 domain
ppp|Physcomitrium_patens_112289355_ERG3-like	C2	4..95(1.4e-15)	PF00168, C2 domain
smo|Selaginella_moellendorffii_SELMODRAFT_148785	C2	4..95(2e-19)	PF00168, C2 domain

smo|Selaginella_moellendorffii_SELMODRAFT_229026	C2	4..95(1.2e-19)	PF00168, C2 domain
atr|Amborella_trichopoda_v1.0_scaffold00009.330	C2	4..96(7.5e-22)	PF00168, C2 domain
atr|Amborella_trichopoda_v1.0_scaffold00009.331	C2	4..95(8.2e-20)	PF00168, C2 domain
spo|Spirodela_polyrhiza_v2_Spipo23G0016400	C2	6..105(1.3e-20)	PF00168, C2 domain
spo|Spirodela_polyrhiza_v2_Spipo23G0016300	C2	6..102(4e-19)	PF00168, C2 domain
spo|Spirodela_polyrhiza_v2_Spipo16G0035500	C2	4..93(2.9e-18)	PF00168, C2 domain
osa|Oryza_sativa_v7.0_LOC_Os01g62430.3_OsERG1_RPP17	C2	5..108(7.5e-22)	PF00168, C2 domain
osa|Oryza_sativa_v7.0_LOC_Os01g62430.3_OsERG1_RPP17	B9-C2	40..96(0.017)	PF07162, Ciliary basal body-associated, B9 protein
osa|Oryza_sativa_v7.0_LOC_Os01g62430.3_OsERG1_RPP17	DOCK-C2	40..81(0.14)	PF14429, C2 domain in Dock180 and Zizimin proteins
osa|Oryza_sativa_v7.0_LOC_Os04g44870.1_OsERG3_RPP16	C2	4..97(3.3e-20)	PF00168, C2 domain
osa|Oryza_sativa_v7.0_LOC_Os04g58570.1	C2	4..93(1.3e-20)	PF00168, C2 domain
osa|Oryza_sativa_v7.0_LOC_Os02g42710.1	C2	4..96(4.8e-19)	PF00168, C2 domain
pvi|Panicum_virgatum_v5.1_Pavir.5KG621600.2.p	C2	4..109(4.7e-21)	PF00168, C2 domain
pvi|Panicum_virgatum_v5.1_Pavir.5KG621600.2.p	PI3K_C2	41..97(0.17)	PF00792, Phosphoinositide 3-kinase C2
pvi|Panicum_virgatum_v5.1_Pavir.5NG582800.1.p	C2	4..109(2.4e-22)	PF00168, C2 domain
pvi|Panicum_virgatum_v5.1_Pavir.5NG582800.1.p	Sec39	15..91(0.018)	PF08314, Secretory pathway protein Sec39
pvi|Panicum_virgatum_v5.1_Pavir.5NG582800.1.p	PI3K_C2	42..96(0.18)	PF00792, Phosphoinositide 3-kinase C2
pvi|Panicum_virgatum_v5.1_Pavir.1NG368400.1.p	C2	4..98(1.4e-19)	PF00168, C2 domain
pvi|Panicum_virgatum_v5.1_Pavir.1KG408900.1.p	C2	4..98(1.4e-19)	PF00168, C2 domain
pvi|Panicum_virgatum_v5.1_Pavir.7NG338700.1.p	C2	4..96(1.1e-19)	PF00168, C2 domain
pvi|Panicum_virgatum_v5.1_Pavir.7NG444500.3.p	C2	4..95(3.1e-20)	PF00168, C2 domain
pvi|Panicum_virgatum_v5.1_Pavir.7KG372100.3.p	C2	4..95(3.7e-20)	PF00168, C2 domain
sbi|Sorghum_bicolor_v3.1.1_Sobic.003G352400.1	C2	4..108(5.9e-22)	PF00168, C2 domain
sbi|Sorghum_bicolor_v3.1.1_Sobic.003G352400.1	DOCK-C2	39..80(0.19)	PF14429, C2 domain in Dock180 and Zizimin proteins
sbi|Sorghum_bicolor_v3.1.1_Sobic.004G228300.1	C2	4..97(3.2e-18)	PF00168, C2 domain
sbi|Sorghum_bicolor_v3.1.1_Sobic.006G157900.1	C2	5..97(4.2e-18)	PF00168, C2 domain
sbi|Sorghum_bicolor_v3.1.1_Sobic.006G271300.1	C2	4..93(3.4e-20)	PF00168, C2 domain
zma|Zea_mays_RefGen_V4_Zm00001d042909_P001	C2	4..109(5.9e-22)	PF00168, C2 domain

zma|Zea_mays_RefGen_V4_Zm00001d042909_P001	Sec39	14..91(0.006)	PF08314, Secretory pathway protein Sec39
zma|Zea_mays_RefGen_V4_Zm00001d042909_P001	DOCK-C2	39..80(0.56)	PF14429, C2 domain in Dock180 and Zizimin proteins
zma|Zea_mays_RefGen_V4_Zm00001d051249_P003	C2	4..97(3.5e-18)	PF00168, C2 domain
zma|Zea_mays_RefGen_V4_Zm00001d002850_P001	C2	4..96(4.5e-18)	PF00168, C2 domain
zma|Zea_mays_RefGen_V4_Zm00001d026642_P001	C2	4..95(3.6e-20)	PF00168, C2 domain
sly|Solanum_lycopersicum_ITAG3.2_Solyc04g011540.3.1	C2	6..102(5.6e-18)	PF00168, C2 domain
sly|Solanum_lycopersicum_ITAG3.2_Solyc04g011540.3.1	Big_3	105..147(0.017)	PF07523, Bacterial Ig-like domain (group 3)
sly|Solanum_lycopersicum_ITAG3.2_Solyc10g018060.2.1	C2	4..105(1.5e-19)	PF00168, C2 domain
sly|Solanum_lycopersicum_ITAG3.2_Solyc08g080680.3.1	C2	4..96(4.8e-21)	PF00168, C2 domain
stu|Solanum_tuberosum_v6.1_Soltu.DM.04G007530.1	C2	6..102(5.5e-18)	PF00168, C2 domain
stu|Solanum_tuberosum_v6.1_Soltu.DM.04G007530.1	Big_3	105..147(0.25)	PF07523, Bacterial Ig-like domain (group 3)
stu|Solanum_tuberosum_v6.1_Soltu.DM.10G007190.1	C2	4..105(4.6e-18)	PF00168, C2 domain
stu|Solanum_tuberosum_v6.1_Soltu.DM.08G027310.1	C2	4..97(8.5e-21)	PF00168, C2 domain
egr|Eucalyptus_grandis_v2.0_Eucgr.J00003.1	C2	5..108(1.9e-16)	PF00168, C2 domain
egr|Eucalyptus_grandis_v2.0_Eucgr.E00562.1	C2	4..95(5.1e-20)	PF00168, C2 domain
vvi|Vitis_vinifera_v2.1_VIT_213s0019g01490.1	C2	5..102(6.8e-20)	PF00168, C2 domain
vvi|Vitis_vinifera_v2.1_VIT_208s0007g00430.1	C2	8..106(3.6e-16)	PF00168, C2 domain
vvi|Vitis_vinifera_v2.1_VIT_202s0025g04350.1	C2	4..96(8.4e-21)	PF00168, C2 domain
pop|Populus_trichocarpa_v4.1_Potri.010G203000.2.p	C2	4..107(5.6e-20)	PF00168, C2 domain
pop|Populus_trichocarpa_v4.1_Potri.010G203100.1.p	C2	4..103(8.8e-20)	PF00168, C2 domain
pop|Populus_trichocarpa_v4.1_Potri.002G155600.1.p	C2	4..94(2.4e-19)	PF00168, C2 domain
rco|Ricinus_communis_v0.1_27383.m000160	C2	4..104(4.1e-20)	PF00168, C2 domain
rco|Ricinus_communis_v0.1_30190.m011166	C2	4..101(3.3e-19)	PF00168, C2 domain
rco|Ricinus_communis_v0.1_30174.m008982	C2	4..94(1.2e-19)	PF00168, C2 domain
csi|Citrus_sinensis_v1.1_orange1.1g035563m_ERG1_CsPP16-1	C2	5..106(6.7e-17)	PF00168, C2 domain
csi|Citrus_sinensis_v1.1_orange1.1g046962m_At1g63220-like_CsPP16-2	C2	4..104(6.6e-21)	PF00168, C2 domain

csi|Citrus_sinensis_v1.1_orange1.1g037768m_ERG3-like_isoform-1_CsPP16-3	C2	4..94(6e-19)	PF00168, C2 domain
ccl|Citrus_clementina_v1.0_Ciclev10032979m	C2	5..106(6.4e-17)	PF00168, C2 domain
ccl|Citrus_clementina_v1.0_Ciclev10024188m	C2	4..104(6.6e-21)	PF00168, C2 domain
ccl|Citrus_clementina_v1.0_Ciclev10022928m	C2	4..41(0.0022)<br>48..78(0.00034)	PF00168, C2 domain
ccl|Citrus_clementina_v1.0_Ciclev10022928m	Npun_R1517	29..67(0.098)	PF18068, Npun R1517
cpa|Carica_papaya_ASGPB_v0.4_1.414	C2	5..102(7.2e-19)	PF00168, C2 domain
cpa|Carica_papaya_ASGPB_v0.4_1.415	C2	3..73(9.5e-11)	PF00168, C2 domain
cpa|Carica_papaya_ASGPB_v0.4_18.207	C2	4..92(7.1e-21)	PF00168, C2 domain
tca|Theobroma_cacao_v2.1_Thecc.10G040600.1.p	C2	4..102(5e-18)	PF00168, C2 domain
tca|Theobroma_cacao_v2.1_Thecc.03G274700.1.p	C2	4..94(2.3e-20)	PF00168, C2 domain
tca|Theobroma_cacao_v2.1_Thecc.01G359300.1.p	C2	4..97(2.1e-12)	PF00168, C2 domain
aly|Arabidopsis_lyrata_v2.1_AL5G36620.t1	C2	4..107(2.9e-21)	PF00168, C2 domain
aly|Arabidopsis_lyrata_v2.1_AL2G12160.t1	C2	4..94(8.6e-19)	PF00168, C2 domain
ath|Arabidopsis_thaliana_TAIR10_AT3G55470.1_AtPP16-1	C2	4..107(2.9e-21)	PF00168, C2 domain
ath|Arabidopsis_thaliana_TAIR10_AT1G63220.1_AtPP16-2	C2	4..94(8.3e-19)	PF00168, C2 domain
bra|Brassica_rapa_FPsc_v1.3_Brara.D00394.1	C2	4..108(1.1e-22)	PF00168, C2 domain
bra|Brassica_rapa_FPsc_v1.3_Brara.I03796.1	C2	4..105(9.3e-20)	PF00168, C2 domain
bra|Brassica_rapa_FPsc_v1.3_Brara.I01220.1	C2	4..94(1.1e-18)	PF00168, C2 domain
esa|Eutrema_salsugineum_v1.0_Thhalv10010786m	C2	4..105(1.4e-19)	PF00168, C2 domain
esa|Eutrema_salsugineum_v1.0_Thhalv10023745m	C2	4..94(2.4e-18)	PF00168, C2 domain
csa|Cucumis_sativus_v1.0_Cucsa.010870.1	C2	4..99(1.7e-19)	PF00168, C2 domain
csa|Cucumis_sativus_v1.0_Cucsa.010880.1	C2	4..100(4.5e-18)	PF00168, C2 domain
csa|Cucumis_sativus_v1.0_Cucsa.302880.1	C2	4..98(2.8e-19)	PF00168, C2 domain
gmx|Glycine_max_Wm82.a4.v1_Glyma.02G247900.3.p	C2	6..105(2e-21)	PF00168, C2 domain
gmx|Glycine_max_Wm82.a4.v1_Glyma.11G219000.1.p	C2	6..102(2.2e-20)	PF00168, C2 domain
gmx|Glycine_max_Wm82.a4.v1_Glyma.11G219000.1.p	PI3K_C2	38..77(0.12)	PF00792, Phosphoinositide 3-kinase C2
gmx|Glycine_max_Wm82.a4.v1_Glyma.18G038400.1.p	C2	6..101(6.7e-21)	PF00168, C2 domain
gmx|Glycine_max_Wm82.a4.v1_Glyma.18G038400.1.p	B9-C2	34..93(0.13)	PF07162, Ciliary basal body-associated, B9 protein
gmx|Glycine_max_Wm82.a4.v1_Glyma.14G068400.1.p	C2	6..103(2.2e-20)	PF00168, C2 domain

gmx|Glycine_max_Wm82.a4.v1_Glyma.02G248000.1.p	C2	6..103(2.4e-20)	PF00168, C2 domain
gmx|Glycine_max_Wm82.a4.v1_Glyma.02G248100.1.p	C2	6..101(3.3e-21)	PF00168, C2 domain
gmx|Glycine_max_Wm82.a4.v1_Glyma.03G015400.1.p	C2	4..97(2.7e-20)	PF00168, C2 domain
gmx|Glycine_max_Wm82.a4.v1_Glyma.07G075300.2.p	C2	4..97(5.2e-20)	PF00168, C2 domain
gmx|Glycine_max_Wm82.a4.v1_Glyma.18G224800.1.p	C2	4..94(2.1e-21)	PF00168, C2 domain
gmx|Glycine_max_Wm82.a4.v1_Glyma.08G011900.1.p	C2	4..93(4.1e-18)	PF00168, C2 domain
gmx|Glycine_max_Wm82.a4.v1_Glyma.05G204900.1.p	C2	4..93(3.2e-18)	PF00168, C2 domain
pvu|Phaseolus_vulgaris_v2.1_Phvul.008G232800.1	C2	6..103(2.5e-20)	PF00168, C2 domain
pvu|Phaseolus_vulgaris_v2.1_Phvul.008G232700.1	C2	6..103(1.2e-21)	PF00168, C2 domain
pvu|Phaseolus_vulgaris_v2.1_Phvul.008G232600.1	C2	7..103(5.4e-19)	PF00168, C2 domain
pvu|Phaseolus_vulgaris_v2.1_Phvul.002G286900.1	C2	4..96(5.8e-21)	PF00168, C2 domain
pvu|Phaseolus_vulgaris_v2.1_Phvul.002G286900.1	BCL9	119..130(0.21)	PF11502, B-cell lymphoma 9 protein
pvu|Phaseolus_vulgaris_v2.1_Phvul.010G093800.1	C2	4..96(2.5e-21)	PF00168, C2 domain
pvu|Phaseolus_vulgaris_v2.1_Phvul.008G073400.1	C2	4..96(1.5e-21)	PF00168, C2 domain
pper|Prunus_persica_v2.1_Prupe.2G236500.1	C2	4..103(2.8e-19)	PF00168, C2 domain
pper|Prunus_persica_v2.1_Prupe.2G236500.1	Mago-bind	117..129(0.18)	PF09282, Mago binding
pper|Prunus_persica_v2.1_Prupe.6G256400.1	C2	4..101(1.4e-17)	PF00168, C2 domain
pper|Prunus_persica_v2.1_Prupe.6G256400.1	Ribos_L4_asso_C	15..38(0.32)	PF14374, 60S ribosomal protein L4 C-terminal domain
pper|Prunus_persica_v2.1_Prupe.2G158500.1	C2	4..95(3.4e-21)	PF00168, C2 domain
pper|Prunus_persica_v2.1_Prupe.5G094600.1	C2	4..96(2.4e-24)	PF00168, C2 domain
pper|Prunus_persica_v2.1_Prupe.2G158700.1	C2	4..96(1.4e-21)	PF00168, C2 domain
pper|Prunus_persica_v2.1_Prupe.2G158800.1	C2	4..97(1e-20)	PF00168, C2 domain
pper|Prunus_persica_v2.1_Prupe.5G094700.1	C2	5..104(2.3e-21)	PF00168, C2 domain
mpp|Micromonas_pusilla_CCMP1545_v3.0_36336	C2	400..490(3e-15)	PF00168, C2 domain
mpp|Micromonas_pusilla_CCMP1545_v3.0_36336	SMP_LBD	36..220(8.4e-13)	PF17047, Synaptotagmin-like mitochondrial-lipid-binding domain
mpp|Micromonas_pusilla_CCMP1545_v3.0_57933	C2	629..734(1.6e-18)<br>803..896(6.1e-16)	PF00168, C2 domain
mpp|Micromonas_pusilla_CCMP1545_v3.0_57933	MMM1	148..228(0.28)	PF10296, Maintenance of mitochondrial morphology protein 1
mpp|Micromonas_pusilla_CCMP1545_v3.0_63907	C2	122..214(3.6e-19)	PF00168, C2 domain

mpp|Micromonas_pusilla_CCMP1545_v3.0_58763	Kelch_4	108..141(0.024)<br>171..223(7e-09)<br>224..276(0.0016)<br>356..389(0.012)	PF13418, Galactose oxidase, central domain
mpp|Micromonas_pusilla_CCMP1545_v3.0_58763	Kelch_3	116..178(0.073)<br>182..232(2.3e-11)<br>243..279(0.071)<br>356..405(0.12)	PF13415, Galactose oxidase, central domain
mpp|Micromonas_pusilla_CCMP1545_v3.0_58763	Kelch_5	107..138(0.0021)<br>169..203(1.4e-05)<br>222..273(5.5e-06)	PF13854, Kelch motif
mpp|Micromonas_pusilla_CCMP1545_v3.0_58763	C2	517..611(1e-16)	PF00168, C2 domain
mpp|Micromonas_pusilla_CCMP1545_v3.0_58763	Kelch_1	108..134(0.73)<br>172..212(1.1e-09)<br>244..276(0.032)	PF01344, Kelch motif
mpp|Micromonas_pusilla_CCMP1545_v3.0_58763	Kelch_6	109..145(0.01)<br>172..212(0.00061)	PF13964, Kelch motif
mpp|Micromonas_pusilla_CCMP1545_v3.0_58763	BTB	853..904(0.11)	PF00651, BTB/POZ domain
mpp|Micromonas_pusilla_CCMP1545_v3.0_58763	Kelch_2	171..212(0.21)	PF07646, Kelch motif
mis|Micromonas_sp_RCC299_v3.0_59753	C2	298..378(1.5e-15)<br>585..679(2.3e-13)	PF00168, C2 domain
mis|Micromonas_sp_RCC299_v3.0_59753	SMP_LBD	95..276(0.00015)	PF17047, Synaptotagmin-like mitochondrial-lipid-binding domain
mis|Micromonas_sp_RCC299_v3.0_59753	DOCK-C2	330..385(0.049)	PF14429, C2 domain in Dock180 and Zizimin proteins
mis|Micromonas_sp_RCC299_v3.0_61618	C2	179..273(1.8e-18)<br>373..470(1.6e-14)<br>527..621(9.3e-07)	PF00168, C2 domain
mis|Micromonas_sp_RCC299_v3.0_61618	PRT_C	896..971(1.2e-05)	PF08372, Plant phosphoribosyltransferase C-terminal
mis|Micromonas_sp_RCC299_v3.0_61618	AAA_11	818..929(0.0012)	PF13086, AAA domain
mis|Micromonas_sp_RCC299_v3.0_61618	DDHD	815..894(0.0095)	PF02862, DDHD domain
mis|Micromonas_sp_RCC299_v3.0_61618	Reticulon	931..981(0.044)	PF02453, Reticulon

mis|Micromonas_sp_RCC299_v3.0_61618	CAF1	793..880(0.23)	PF04857, CAF1 family ribonuclease
mis|Micromonas_sp_RCC299_v3.0_61618	Pex24p	928..987(0.058)	PF06398, Integral peroxisomal membrane peroxin
mis|Micromonas_sp_RCC299_v3.0_61618	DUF5427	809..934(0.83)	PF10310, Family of unknown function (DUF5427)
mis|Micromonas_sp_RCC299_v3.0_59622	C2	625..736(1.7e-10)	PF00168, C2 domain
mis|Micromonas_sp_RCC299_v3.0_59622	Exo_endo_phos	109..511(3e-06)	PF03372, Endonuclease/Exonuclease/phosphatase family
mis|Micromonas_sp_RCC299_v3.0_59622	AvrPto	523..606(0.33)	PF11592, Central core of the bacterial effector protein AvrPto
olu|Ostreococcus_lucimarinus_v2.0_8959	C2	18..114(1.4e-21)	PF00168, C2 domain
olu|Ostreococcus_lucimarinus_v2.0_9049	C2	1..95(2.4e-16)	PF00168, C2 domain
csl|Coccomyxa_subellipsoidea_C-169_v2.0_63427	C2	346..447(7.8e-19)	PF00168, C2 domain
csl|Coccomyxa_subellipsoidea_C-169_v2.0_63427	SMP_LBD	114..177(0.64) 249..332(0.00032)	PF17047, Synaptotagmin-like mitochondrial-lipid-binding domain
csl|Coccomyxa_subellipsoidea_C-169_v2.0_56339	Nucleopor_Nup85	53..181(6.5e-11) 235..437(2.3e-21) 460..636(3.6e-13)	PF07575, Nup85 Nucleoporin
csl|Coccomyxa_subellipsoidea_C-169_v2.0_56339	Lipase_3	845..1037(3e-27)	PF01764, Lipase (class 3)
csl|Coccomyxa_subellipsoidea_C-169_v2.0_56339	C2	672..750(2.1e-18)	PF00168, C2 domain
csl|Coccomyxa_subellipsoidea_C-169_v2.0_56339	DUF2428	1073..1132(0.33)	PF10350, THADA/TRM732, DUF2428
vca|Volvox_carteri_v2.1_Vocar.0007s0408.1.p	C2	6..97(6.5e-16)	PF00168, C2 domain
vca|Volvox_carteri_v2.1_Vocar.0013s0032.1.p	C2	6..95(1.5e-20)	PF00168, C2 domain
vca|Volvox_carteri_v2.1_Vocar.0009s0235.1.p	C2	6..102(1.3e-14)	PF00168, C2 domain
vca|Volvox_carteri_v2.1_Vocar.0001s1625.1.p	C2	7..92(9.4e-18)	PF00168, C2 domain
vca|Volvox_carteri_v2.1_Vocar.0015s0039.1.p	C2	9..102(1.3e-16)	PF00168, C2 domain
vca|Volvox_carteri_v2.1_Vocar.0009s0192.1.p	C2	8..96(1.6e-17)	PF00168, C2 domain
vca|Volvox_carteri_v2.1_Vocar.0022s0215.1.p	C2	8..98(7.7e-16)	PF00168, C2 domain
vca|Volvox_carteri_v2.1_Vocar.0004s0220.1.p	Pkinase	170..428(4.4e-77)	PF00069, Protein kinase domain
vca|Volvox_carteri_v2.1_Vocar.0004s0220.1.p	PK_Tyr_Ser-Thr	174..424(6.5e-34)	PF07714, Protein tyrosine and serine/threonine kinase

vca|Volvox_carteri_v2.1_Vocar.0004s0220.1.p	EF-hand_1	476..502(7e-07)<br>513..536(4e-06)<br>549..574(6.3e-06)<br>584..612(1.2e-06)	PF00036, EF hand
vca|Volvox_carteri_v2.1_Vocar.0004s0220.1.p	EF-hand_7	476..537(6.4e-10)<br>547..610(2.1e-11)	PF13499, EF-hand domain pair
vca|Volvox_carteri_v2.1_Vocar.0004s0220.1.p	EF-hand_6	476..502(6.2e-06)<br>520..536(0.27)<br>549..575(6.5e-06)<br>585..610(0.022)	PF13405, EF-hand domain
vca|Volvox_carteri_v2.1_Vocar.0004s0220.1.p	EF-hand_5	477..497(0.00043)<br>517..533(0.00033)<br>549..569(3.8e-05)<br>585..609(0.015)	PF13202, EF hand
vca|Volvox_carteri_v2.1_Vocar.0004s0220.1.p	EF-hand_8	476..502(0.0052)<br>488..535(1.3e-10)<br>561..610(4e-08)	PF13833, EF-hand domain pair
vca|Volvox_carteri_v2.1_Vocar.0004s0220.1.p	C2	6..105(3.6e-11)	PF00168, C2 domain
vca|Volvox_carteri_v2.1_Vocar.0004s0220.1.p	Kinase-like	263..380(0.00014)	PF14531, Kinase-like
vca|Volvox_carteri_v2.1_Vocar.0004s0220.1.p	EF-hand_11	480..536(0.00018)	PF08976, EF-hand domain
vca|Volvox_carteri_v2.1_Vocar.0004s0220.1.p	EF-hand_4	472..535(0.012)	PF12763, Cytoskeletal-regulatory complex EF hand
vca|Volvox_carteri_v2.1_Vocar.0004s0220.1.p	EF-hand_9	483..535(0.0077)	PF14658, EF-hand domain
vca|Volvox_carteri_v2.1_Vocar.0004s0220.1.p	Kdo	261..306(0.025)	PF06293, Lipopolysaccharide kinase (Kdo/WaaP) family
cre|Chlamydomonas_reinhardtii_v5.6_Cre01.g015500.t1.2	C2	6..97(4.3e-20)	PF00168, C2 domain
cre|Chlamydomonas_reinhardtii_v5.6_Cre02.g110350.t1.2	C2	6..100(2.1e-20)	PF00168, C2 domain
cre|Chlamydomonas_reinhardtii_v5.6_Cre17.g723700.t1.1	C2	7..93(2.5e-20)	PF00168, C2 domain
cre|Chlamydomonas_reinhardtii_v5.6_Cre12.g485200.t1.1	C2	8..92(2.6e-20)	PF00168, C2 domain
cre|Chlamydomonas_reinhardtii_v5.6_Cre11.g467599.t1.1	C2	8..95(3.9e-19)	PF00168, C2 domain
cre|Chlamydomonas_reinhardtii_v5.6_Cre11.g467594.t1.1	C2	8..95(3.9e-19)	PF00168, C2 domain
cre|Chlamydomonas_reinhardtii_v5.6_Cre11.g467593.t1.1	C2	8..95(8.6e-20)	PF00168, C2 domain
cre|Chlamydomonas_reinhardtii_v5.6_Cre11.g467601.t1.1	C2	8..94(2.2e-19)	PF00168, C2 domain

cre|Chlamydomonas_reinhardtii_v5.6_Cre11.g467595.t1.1	C2	6..104(6.7e-18)	PF00168, C2 domain
cre|Chlamydomonas_reinhardtii_v5.6_Cre11.g467595.t1.1	TFIIA	129..191(0.0015)	PF03153, Transcription factor IIA, alpha/beta subunit
cre|Chlamydomonas_reinhardtii_v5.6_Cre11.g467595.t1.1	baeRF_family7	167..208(0.08)	PF18849, Bacterial archaeo-eukaryotic release factor family 7
cre|Chlamydomonas_reinhardtii_v5.6_Cre11.g467595.t1.1	Glypican	137..189(0.067)	PF01153, Glypican
cre|Chlamydomonas_reinhardtii_v5.6_Cre06.g305750.t1.2	C2	8..93(5.2e-19)	PF00168, C2 domain
cre|Chlamydomonas_reinhardtii_v5.6_Cre02.g074900.t1.1	C2	530..629(2.6e-13)	PF00168, C2 domain
cre|Chlamydomonas_reinhardtii_v5.6_Cre02.g074900.t1.1	MMM1	282..358(0.49)	PF10296, Maintenance of mitochondrial morphology protein 1

Figure S1. Alignment of pumpkin CmPP16 and PP16 related proteins in green algae species. Clustal Omega version 1.2.3 in local mode was used for sequence alignment and the color code was applied with Jalview version 1.0 software. C2-domain in the alignment is indicated in red.

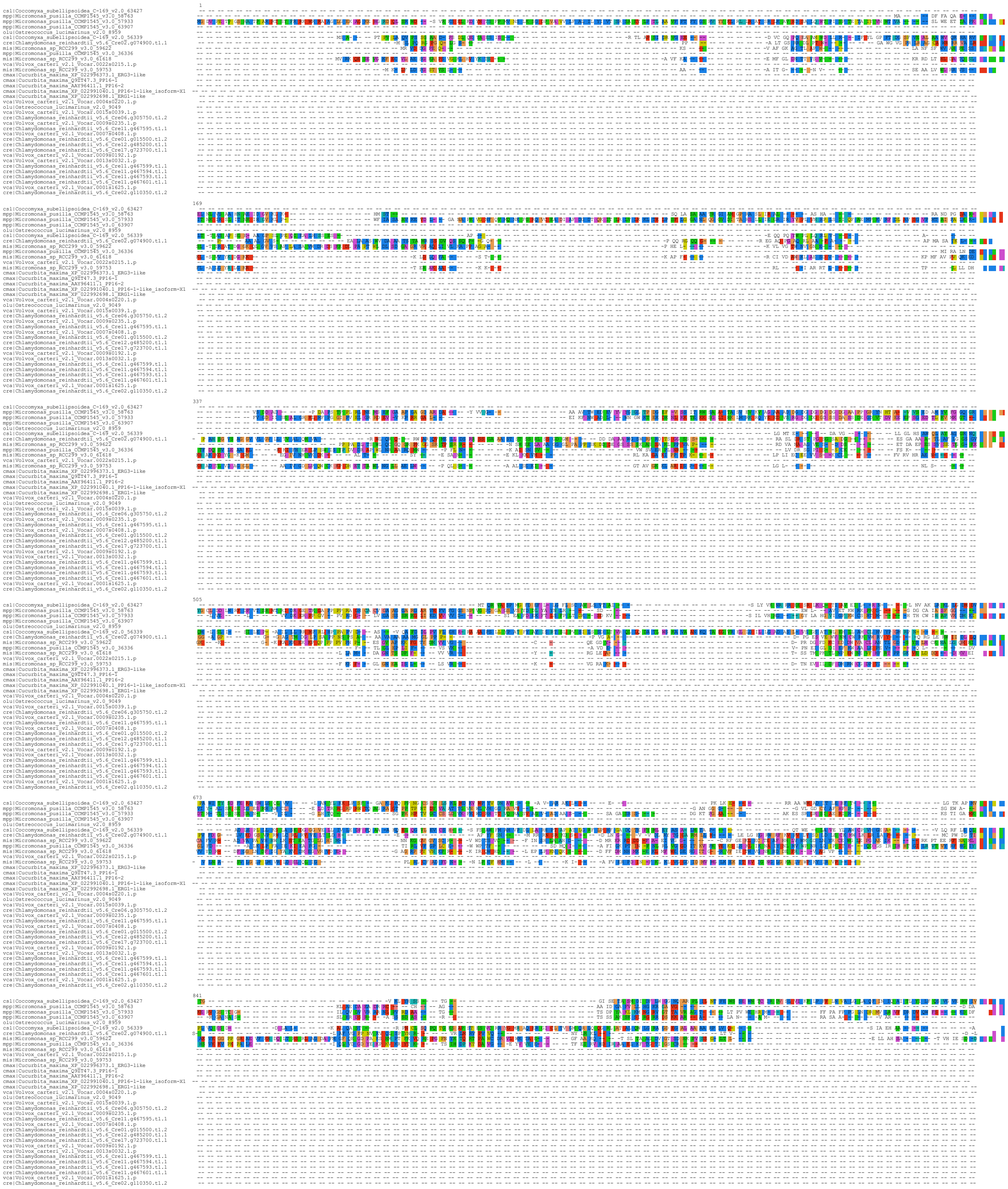

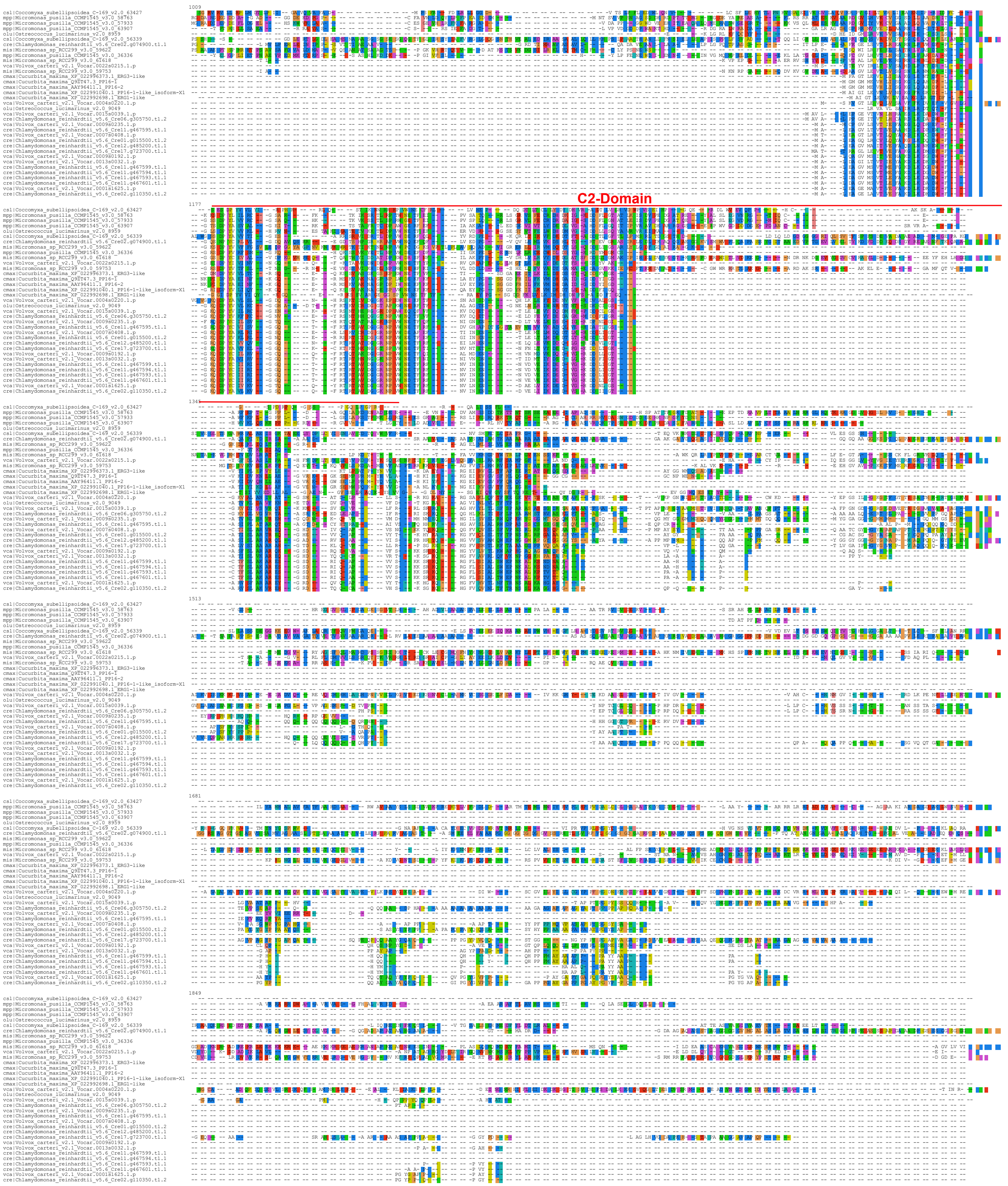
C2-Domain

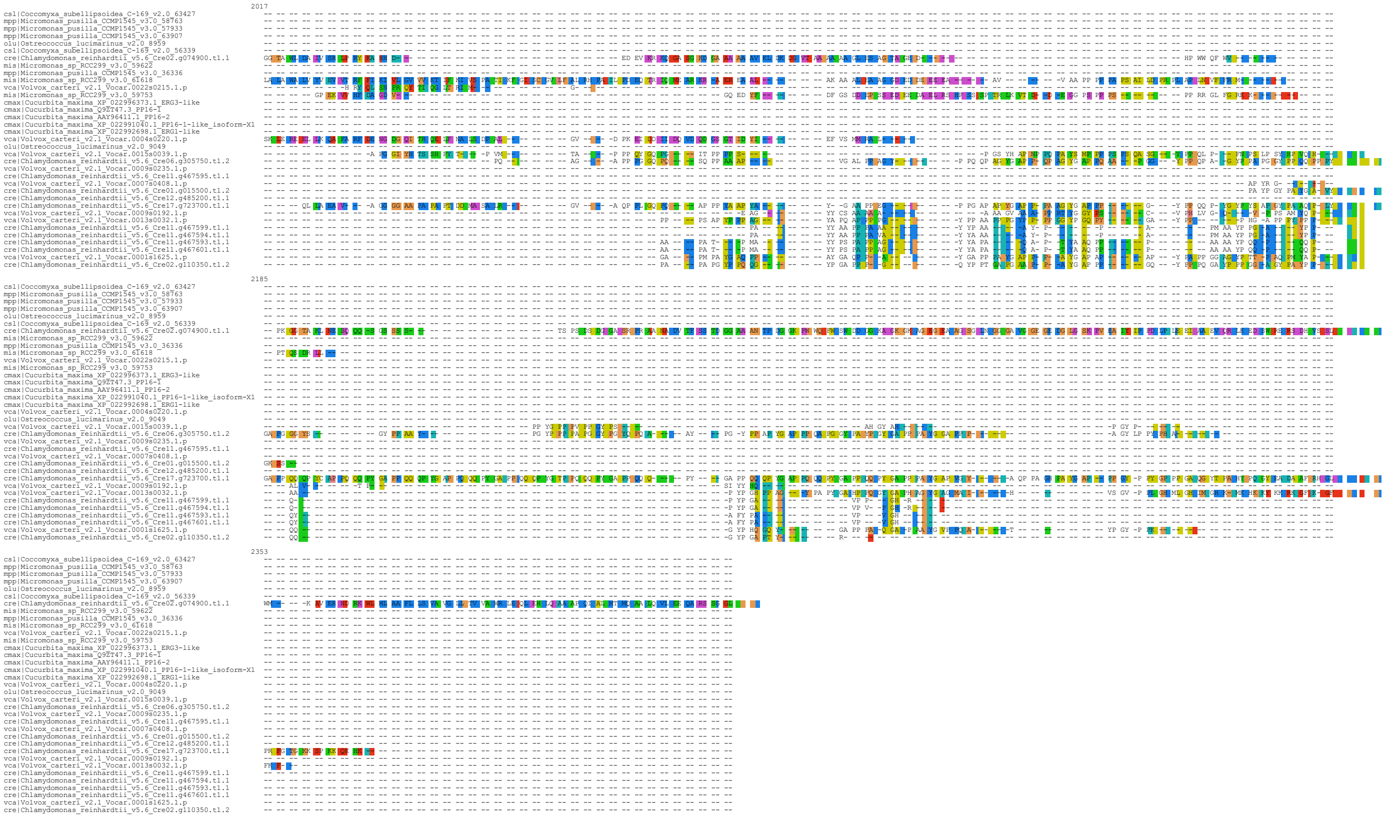
2017
2185
2353
csl|Coccomyxa_subellipsoidea_C-169_v2.0_63427
mpp|Micromonas_pusilla_CCMP1545_v3.0_58763
mpp|Micromonas_pusilla_CCMP1545_v3.0_57933
mpp|Micromonas_pusilla_CCMP1545_v3.0_63907
olu|Ostreococcus_lucimarinus_v2.0_8959
csl|Coccomyxa_subellipsoidea_C-169_v2.0_56339
cre|Chlamydomonas_reinhardtii_v5.6_Cre02.g074900.t1.1
mis|Micromonas_sp_RCC299_v3.0_59622
mpp|Micromonas_pusilla_CCMP1545_v3.0_36336
mis|Micromonas_sp_RCC299_v3.0_61618
vca|Volvox_carteri_v2.1_Vocar.0022s0215.1.p
mis|Micromonas_sp_RCC299_v3.0_59753
cmax|Cucurbita_maxima_XP_022996373.1_ERG3-like
cmax|Cucurbita_maxima_Q9ST47.3_PP16-1
cmax|Cucurbita_maxima_AAY96411.1_PP16-2
cmax|Cucurbita_maxima_XP_022991040.1_PP16-1-like_isoform-X1
cmax|Cucurbita_maxima_XP_022992698.1_ERG1-like
vca|Volvox_carteri_v2.1_Vocar.0004s0220.1.p
olu|Ostreococcus_lucimarinus_v2.0_9049
vca|Volvox_carteri_v2.1_Vocar.0015s0039.1.p
cre|Chlamydomonas_reinhardtii_v5.6_Cre06.g305750.t1.2
vca|Volvox_carteri_v2.1_Vocar.0009s0235.1.p
cre|Chlamydomonas_reinhardtii_v5.6_Cre11.g467595.t1.1
vca|Volvox_carteri_v2.1_Vocar.0007s0408.1.p
cre|Chlamydomonas_reinhardtii_v5.6_Cre01.g015500.t1.2
cre|Chlamydomonas_reinhardtii_v5.6_Cre12.g485200.t1.1
cre|Chlamydomonas_reinhardtii_v5.6_Cre17.g723700.t1.1
vca|Volvox_carteri_v2.1_Vocar.0009s0192.1.p
vca|Volvox_carteri_v2.1_Vocar.0013s0032.1.p
cre|Chlamydomonas_reinhardtii_v5.6_Cre11.g467599.t1.1
cre|Chlamydomonas_reinhardtii_v5.6_Cre11.g467594.t1.1
cre|Chlamydomonas_reinhardtii_v5.6_Cre11.g467593.t1.1
cre|Chlamydomonas_reinhardtii_v5.6_Cre11.g467601.t1.1
vca|Volvox_carteri_v2.1_Vocar.0001s1625.1.p
cre|Chlamydomonas_reinhardtii_v5.6_Cre02.g110350.t1.2
